# An Electrical Model of Hydrocephalus Shunt Incorporating the CSF Dynamics

**DOI:** 10.1038/s41598-019-46328-z

**Published:** 2019-07-05

**Authors:** R. Baghbani

**Affiliations:** Biomedical Engineering Department, Hamedan University of Technology, Hamedan, Iran

**Keywords:** Neurophysiology, Biomedical engineering

## Abstract

The accumulation of cerebrospinal fluid (CSF) in brain ventricles and subarachnoid space is known as hydrocephalus. Hydrocephalus is a result of disturbances in the secretion or absorption process of CSF. A hydrocephalus shunt is an effective method for the treatment of hydrocephalus. In this paper, at first, the procedures of secretion, circulation, and absorption of CSF are studied and subsequently, the mathematical relations governing the pressures in different interacting compartments of the brain are considered. A mechanical-electrical model is suggested based on the brain physiology and blood circulation. In the proposed model, hydrocephalus is modeled with an incremental resistance (*R*_*o*_) and hydrocephalus shunt, which is a low resistance path to drain the accumulated CSF in the brain ventricles, is modeled with a resistance in series with a diode. At the end, the simulation results are shown. The simulation results can be used to predict the shunt efficiency in reducing CSF pressure and before a real shunt implementation surgery is carried out in a patient’s body.

## Introduction

Cerebrospinal fluid (CSF) is a clear and colorless fluid that contains small quantities of glucose and protein. CSF fills the brain ventricles and the central canal of the spinal cord^1^. It is mainly produced in the ependymal surfaces of the Choroid plexus, and passes through the third and fourth ventricles by diffusion. It then arrives in the subarachnoid space and circulates around the brain and spinal cord. The accumulated CSF in the subarachnoid space is mainly drained in the superior sagittal sinus by arachnoid granulations. Hydrocephalus is generally defined as an excessive accumulation of CSF in subarachnoid space and brain ventricles and often results in increasing intracranial pressure (ICP)^2^. This accumulation causes high pressure difference above 200 mmHg and leads to serious problems in central nervous system operation.

Depending on the physiological conditions, hydrocephalus is classified into two types: communicating and non-communicating hydrocephalus. When the flow of CSF is blocked along the narrow channels between ventricles, non-communicating or obstructive hydrocephalus occurs. Communicating or non-obstructive hydrocephalus occurs when the flow of CSF is blocked after passing through the brain ventricles^3^. Communicating hydrocephalus is the most conventional type of hydrocephalus and occurs due to the impaired absorption of CSF without any obstruction in the CSF flow through the brain ventricles and subarachnoid space. It is theorized as an impaired function of arachnoid granulations, which are placed in the superior sagittal sinus.

Arachnoid granulations are the places where CSF is mainly absorbed and returns to venous blood circulation. Several neurological conditions such as intra-ventricular/subarachnoid hemorrhage, meningitis and congenital absence of the arachnoid villi would result in communicating hydrocephalus^4^.

## Physiology Background

### CSF production

CSF is produced by active secretion from cerebral arterial blood^5–7^. CSF is mainly, but not solely, produced by the choroid plexuses^8^. According to^9^, ependymal cells of the ventricles (ependyma) are other sources of CSF production. Capillaries in blood-brain barrier generate CSF, but in a very small value^5,9^. The site of this procedure is only conceptually limited to the choroid plexus of the brain ventricles. Extra-choroidal production of CSF is likely accounted for a sufficient amount of the total fluid formation in human body^10^. The production rate of CSF in normal conditions is reported to be fixed. Due to the lack of a direct approach to measure CSF production in short time durations, CSF secretion dynamics have not been reported correctly. Generally, the average secretion rate of CSF is about 0.35 *ml*/min^11^ and remains proportional to the brain metabolism rate.

### CSF circulation

CSF, which is mainly secreted in the lateral and third ventricles, flows through the Sylvius aqueduct and arrives to the fourth ventricle. The passage through this small aperture is done rapidly and its pulsatile nature is detectable by strong dynamic MRI methods^12^. It is believed that this pulsatile nature has no diagnostic significance^13–15^. CSF then moves out of the fourth ventricle through the Magendie foramen and lateral foramina of Luschka into the subarachnoid space. Afterwards, CSF flows upward to the superior sagittal sinus where it is mostly absorbed. CSF partially flows downward into the lumbar subarachnoid space. For the systolic phase of cardiac cycle, CSF moves into the spinal canal, and returns to the cranial compartment during diastole phase^16^.

Accurate circulation of CSF is one of the basic mechanisms that is required for the proper operation of the central nervous system (CNS). Brain and spinal cord float in CSF, as a result, these structures would be less prone to damage in case of mechanical shocks. The free circulation of CSF results in a constant intracranial pressure. The obstruction of CSF flow between the third and fourth ventricles causes the CSF accumulation in the lateral and third ventricles. This problem is known as non-communicating (obstructive) hydrocephalus. The most causes of the obstructive hydrocephalus are congenital stenosis of the Sylvian aqueduct and neoplasms blocking the foramina. The preferred remedy for this condition is ventriculostomy in which a foramen is developed in the floor of the third ventricle until CSF can flow out^17^.

In patients with communicating hydrocephalus in which impaired flow of CSF is occurred in subarachnoid space, ventriculostomy could not be successful and shunting (placing a tube in the brain ventricle to move out the extra CSF into another place of body) is a better treatment.

### CSF absorption

CSF is continuously secreted and absorbed in the entire CSF system due to the filtration and reabsorption of the water content through the capillary walls into the circumfluent brain tissue^8^. In humans, the drainage of CSF into venous system is mainly performed through arachnoid granulations. It should be noted that the inverse transport through these granulations is not possible, i.e. the drainage of CSF into the venous system is stopped if arachnoid ICP pressure becomes less than sagittal sinus pressure. The drainage of CSF into the venous system has a linear nature, i.e. the amount of CSF drainage is proportional to the pressure difference between CSF in arachnoid granulations and sagittal sinus. The inverse of the proportionality coefficient is called resistance to CSF outflow (*R*_*o*_) and it ranges from 6 to 10 *mmHg*/*ml*/min or 800 to 1333 *Pa* in normal subjects^11,18^. On the other hand, in the proposed electrical model R_o_ = 1 Ω is analogous to normal conditions. Therefore, every 1 Ω in the electrical model is approximately equal to 8 mmHg/ml/min. There are various nonlinear theories that describe the reduction in *R*_*o*_ when the CSF drainage rate increases. All of these theories are mainly based on animal models^19^ or supported by measurement methods that are not very accurate^20^. Reducing *R*_*o*_ prevents the rise of ICP when CSF pressure increases.

The spinal cord contribution to the overall cranial/spinal compliance is a known phenomenon^21^; however, it is generally assumed that the spinal CSF space has a little role in the CSF drainage in comparison with the cranial compartment. Although spinal CSF transport has not been quantified directly, anatomic evidences reveal that there are several opportunities for the CSF drainage out of the spinal subarachnoid compartment^22^.

### Hydrocephalus

Hydrocephalus usually occurs due to the obstruction of CSF outflow in the brain ventricles or in subarachnoid space over the brain. Hydrocephalus could be a result of CSF overproduction, congenital malformation in arachnoid granulations, serious head damages or brain infections. Bleeding in the subarachnoid space may also block the backward path of CSF into the circulatory system.

Brain compression due to the accumulated CSF in ventricles may result in neurological symptoms such as epileptic, convulsion, or mental retardation seizures. Common features of hydrocephalus resulting in raised ICP are headaches, vomiting, and disturbances of consciousness. If the foramina of brain ventricles are blocked, CSF will be accumulated in the ventricles. This situation is called non-communicating hydrocephalus^23^.

The secretion of CSF continues even if the normal pathways are blocked. Consequently, fluid produced in the brain creates a pressure, which causes dilation of brain ventricles and shrinks the brain tissue^8,24–27^. The contraction of the brain tissue often leads to irreversible brain damage. Aqueducts between the brain ventricles may be blocked at birth or after that because of a growing tumor in the brainstem.

Communicating hydrocephalus can be successfully treated by placing a thin tube (shunt) between the brain ventricles and the abdominal cavity or the right atrium during a surgical operation. Excess fluid accumulated in the brain is directed through the narrow tube into the peritoneal cavity or to the right atrium. The shunt placement may involve the risk of infection. The shunt eventually needs to be replaced when the patient grows up.

A remedy to hydrocephalus is a surgical procedure in which a ventricular catheter (a thin tube made of silicon rubber) is placed within the ventricles of the brain to bypass the blockage of CSF outflow and drain excess CSF into the other body cavities where is absorbed in the blood circulation. Most shunts drain CSF into the peritoneal cavity (Ventriculo-Peritoneal shunt or VP shunt), but there are alternative locations such as the right atrium (Ventriculo-Atrial shunt or VA shunt), the pleural cavity (Ventriculo-Pleural shunt or VPL shunt) and gallbladder^28–31^. A shunt system could also be placed in the lumbar spine to redirect the CSF into the peritoneal cavity (Lumbar-peritoneal shunt or LP shunt)^32^. An alternative treatment for the obstructive hydrocephalus in some patients is endoscopic third ventriculostomy or ETV in which a surgically created hole in the floor of the third ventricle permits the CSF to flow directly to the basal cisterns and thereby shortcutting any obstruction^17^.

A complex problem in using an intracranial shunt in hydrocephalus treatment is the occlusion of the ventricular catheter due to the cellular ingrowth^33–35^. A significant amount of current literature on new shunt design focuses on refining shunt material and port orientation to prevent the occlusion or the cellular ingrowth leading to the shunt failure^33,34,36^. The failure rate for all hydrocephalus shunts is about 40% at the first year and 50% at the second year after the shunt insertion^33,36^.

In this paper, we propose an electrical model for hydrocephalus shunt that can be used to predict shunt efficiency. In our model, we model a hydrocephalus shunt by a resistance in series with a diode. We can combine shunt parameters such as lumen diameter, material properties, and valve pressure by a resistance and a diode. This model can potentially be used to test hydrocephalus catheters prior to the *in-vivo* surgical procedure.

## Material and Methods

In this study, we introduce an electrical model, which represents the relationship between blood, CSF, and hydrocephalus shunt. This analytical model is obtained from mechanical and biological structures in the human body.

### Pressure-volume compartmental model

**Figure 1:**
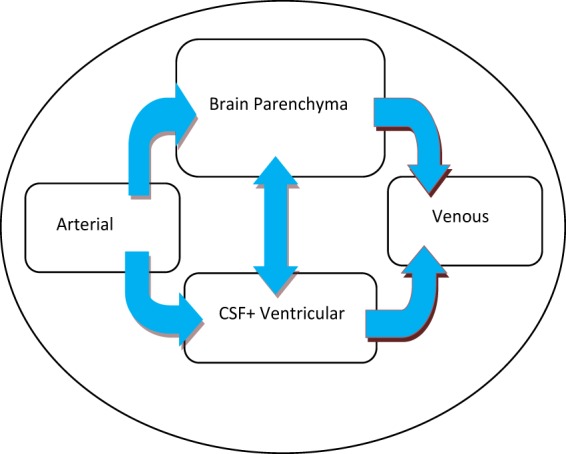
Diagram of the compartmental model that conceptually represents the relationship between volumes and pressures in the human brain.

Pressure-volume compartmental model represents the interaction of pressures and volumes in the brain. The four components of the model are arterial volume, venous volume, brain parenchyma volume and ventricular CSF volume. The arterial volume provides blood and oxygen to the brain system through the arteries. The venous volume includes blood and other substances in the brain that leave the brain and return to the heart. The brain parenchyma volume is composed of real brain tissue (nerves and extracellular fluid) and brain capillary system. The ventricular CSF volume includes CSF volume inside the ventricular system and the entire space occupied by the ventricular system^37^. Compartmental volume-pressure model is conceptually shown in Fig. 1.

### Pressure theory

**Figure 2:**
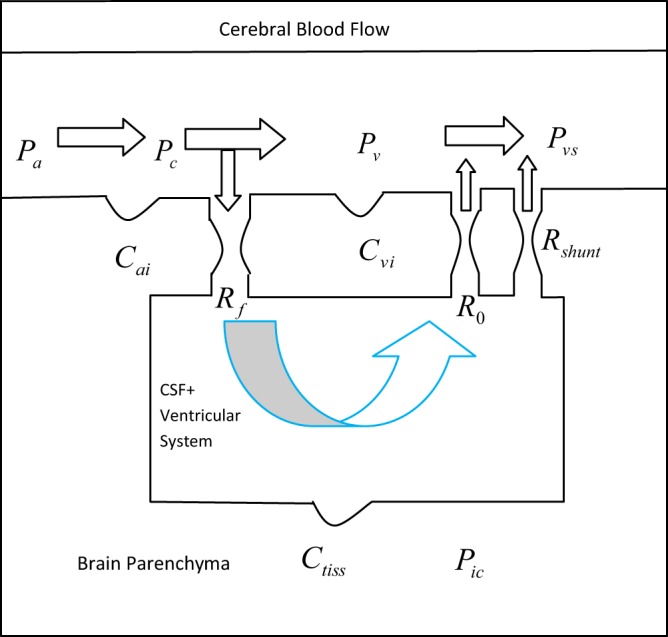
A model of production, circulation, and absorption of CSF fluid along with hydrocephalus shunt.

Blood circulation in the brain from arteries to other parts such as capillaries and veins is shown in Fig. 2. In this representation, the blood flows from the arteries to the capillaries and then to veins. The left vertical pipe and tank are indicative of the ventricular system. CSF heads downward to the tank (*R*_*f*_). It refers to the site of CSF production in choroid plexus in the brain ventricles. CSF surrounds the brain and then moves down to reach to the subarachnoid space.

**Figure 3:**
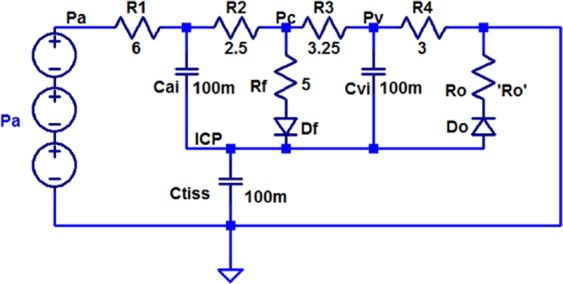
Electrical equivalent circuit of production and circulation of CSF in the brain^37,42^.

CSF is mainly absorbed through the venous system in the sagittal sinus. This path is usually modeled by resistance *R*_*o*_ in Fig. 2. In the model, capacitors represent the tissue compliances. These capacitors model the elasticity of arteries, veins and brain parenchymal tissue. In patients with hydrocephalus, the CSF absorption into the veins in the sagittal sinus is disturbed (resistance *R*_*o*_ increases). In a shunt surgery for hydrocephalus treatment, *R*_*o*_ is actually bypassed and a low-resistance path (*R*_*shunt*_), in its parallel, is created to drain the excess CSF that has accumulated in the ventricles of the brain into the right atrium or peritoneal cavity. Electrical equivalent circuit of Fig. 2 along with the corresponding electrical elements has been shown in Fig. 3.

To obtain various pressures in the compartmental model, at first, an electrical model corresponding to different parts of the brain system and their communication with each other is extracted using Simulink software^37,38^. Secondly, two sine wave with frequency of 1 Hz and 2 Hz are subtracted to obtain a pressure waveform similar to arterial blood pressure in the brain^39^. Thus, the sinusoidal waveform for the pressure within the brain is achieved as follows:

1 $${P}_{a}=17.\,\sin (2\pi t-\frac{\pi }{2})-12.5\,\sin (4\pi t)+16000$$

In the equivalent circuit shown in Fig. 3, all three components of this waveform are modeled with independent voltage sources. The values of the elements along with definition of them are shown in Table 1.

**Table 1:** Value of elements used in the electrical equivalent circuit of Figs 3 and 4.

Element	Description	Value	Reference
R_1_	Tuning pressure waveform resistance	6 Ω	^37, 38^
R_2_	Arterial resistance	2.5 Ω	^37, 38^
R_3_	Capillary resistance	3.25 Ω	^37, 38^
R_4_	Venous resistance	3 Ω	^37, 38^
R_f_	Resistance between arterial blood and choroid plexus	5 Ω	^37, 38^
R_o_	Resistance of arachnoid villi cells	1 Ω	^42^
C_ai_	Compliance between arterials and intracranial site	100 mF	^37^
C_vi_	Compliance between veins and intracranial site	100 mF	^54^
C_tiss_	Compliance of the brain tissue	100 mF	^54^
D_f_	Model of unidirectional secretion of CSF from choroid plexus into the brain ventricles	—	^38, 54^
D_o_	Model of unidirectional drainage of CSF into sagittal sinus	—	^54^
R_shunt_	Resistance of hydrocephalus shunt	1.11 Ω	This work
D_shunt_	Model of unidirectional drainage of hydrocephalus shunt from the brain ventricles into the other place of body	—	This work

### Volume theory

With the pressures shown in Fig. 2, the volume of different parts of the brain could be obtained. With a mass balance for four components of the model, we can write:

2 $$\frac{d{V}_{a}}{dt}+\frac{d{V}_{v}}{dt}+\frac{d{V}_{tiss}}{dt}+\frac{d{V}_{CSF}}{dt}=0$$

where, *a*, *v*, *tiss*, and *CSF* refer to the arterial system, venous system, brain parenchyma and ventricular system volumes of the brain, respectively^40^. The above equation means that changes in the brain volume is zero, i.e. brain volume is fixed. In other words, a part of the brain tissue can enlarge in size (e.g. cerebral ventricles), only if another part compresses.

The volume of separate compartments of the brain is described with an equation that expresses the human brain intracranial dynamics:

3 $$\frac{d{V}_{a}}{dt}={C}_{ai}\frac{d}{dt}({P}_{a}-{P}_{ic})$$

4 $$\frac{d{V}_{v}}{dt}={C}_{vi}\frac{d}{dt}({P}_{v}-{P}_{ic})$$

5 $$\frac{d{V}_{tiss}}{dt}=-\,{C}_{tiss}\frac{d{P}_{ic}}{dt}$$

6 $$\frac{d{V}_{CSF}}{dt}=\frac{{P}_{c}-{P}_{ic}}{{R}_{f}}-\frac{{P}_{ic}-{P}_{vs}}{{R}_{0}\parallel {R}_{shunt}}$$

where, *C*_*ai*_ represents the compliance between arterial compartment and intracranial site, *C*_*vi*_ represents the compliance between venous compartment and intracranial site, and *C*_*tiss*_ represents compliance of the brain tissue. Also, *P*_*a*_, *P*_*c*_, *P*_*v*_, *P*_*ic*_, and *P*_*vs*_ are arterial pressure, capillary pressure, venous pressure, intracranial pressure and pressure difference between arachnoid villi and sagittal sinus, respectively.

**Figure 4:**
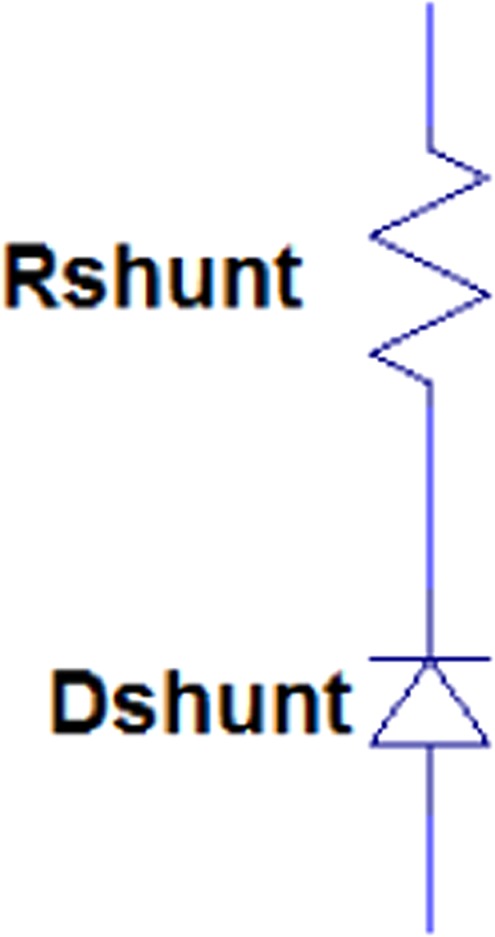
Electrical equivalent circuit of a hydrocephalus shunt.

The circuit shown in Fig. 4 is used to study the effects of hydrocephalus shunt on reducing intracranial pressure. In this paper, we model the hydrocephalus shunt by a resistance in series with a diode. The diode depicts the unidirectional drainage of CSF. We know that Newtonian fluid flow through a tube assimilates an electronic current flow in a resistor in which the fluid flow is analogous to electrical current and the fluid pressure to voltage. Therefore, the equations relating resistive fluid flow through a tube are:

7 $$\begin{array}{lll}{\rm{\Delta }}P=qR & or & R=\frac{{\rm{\Delta }}P}{q}\end{array}$$

where *P* symbolizes the pressure and *q* represents the flow rate in *mL*/*sec*. For laminar flow in a tube, Poiseuille’s law for resistance states that^41^:

8 $$R=\frac{{\rm{\Delta }}P}{q}=\frac{8\mu l}{\pi {r}^{4}}$$

where *μ* is the Newton viscosity, *l* is the length of the tube, and *r* is the inner radius of the tube. In other words, resistance in the fluid flow results from physical aspects of the tube and the fluid.

Therefore, various shunt configurations with different parameters such as lumen diameter, material properties, port configuration, and valve pressure could be considered in the resistance *R*_*shunt*_.

**Figure 5:**
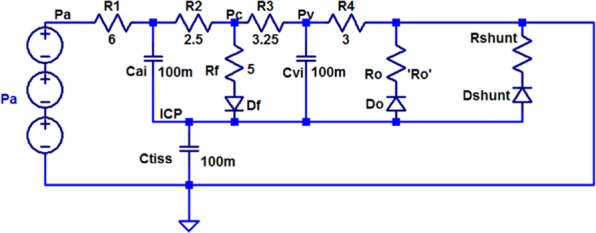
Electrical equivalent circuit of production and circulation of CSF fluid in the brain with hydrocephalus shunt.

We add this model to the electric circuit in Fig. 3 to achieve a complete model of production and circulation dynamics of CSF along with the hydrocephalus shunt (Fig. 5). Using the pressure (voltage) waveforms generated from the circuit under normal and hydrocephalus conditions, behavior of the CSF fluid can be observed and predicted. The output voltage in the circuit in terms of volt is analogous to the brain pressure in terms of Pascal or mmHg. Initially, the circuit was simulated in the normal mode with *R*_*o*_ = 1 Ω (8 mmHg/ml/min). Then the hydrocephalus conditions were simulated for various degrees of hydrocephalus, i.e. moderate hydrocephalus *R*_*o*_ = 5 Ω (40 mmHg/ml/min) and severe hydrocephalus *R*_*o*_ = 10 Ω (80 mmHg/ml/min). In Fig. 5, the CSF drainage resistance into sagittal sinus in the state of severe hydrocephalus is set to 10 Ω (80 mmHg/ml/min). This amount has been chosen based on the fact that the absorption resistance of CSF in patients with hydrocephalus increases by 10 times^42^.

The pressure waveforms in different parts of the brain follow the pulsatile nature of the blood pressure. Since each of these waveforms relates to different places in the brain, they will vary in amplitude and phase^42^. Arterial pressure (*P*_*a*_) is the largest and closest waveform to the heart source. The next largest waveform is capillary pressure *P*_*c*_. As pressure waveform propagates through the brain, it travels from arteries to capillaries. As it is clear from the graphs, when pressure waveform goes away the arteries, it will be significantly weakened^43^. Elasticity of arteries walls absorbs kinetic energy of pressure waveform and decreases its magnitude. After passing through the capillaries, blood goes into the venous system where pressure waveform is further weakened *P*_*v*_. Here blood in the brain returns to the heart and lungs for recycling and purification^43^.

When hydrocephalus occurs, the magnitude of pressure waveforms varies (except for *P*_*a*_) depending on the value of absorption resistance. Hydrocephalus is simulated with increased resistance in the way of absorption of CSF from the ventricular system to its entry into the venous system. In the electrical circuit shown in Fig. 5, the arterial waveform will not be affected by the hydrocephalus. This is physiologically felt, because arterial pressure is not affected by the blockage of the CSF and only depends on the heart function. On the other hand, pressure waveform in the capillary and venous compartments is strongly affected by changing from normal to hydrocephalus conditions. In the case of hydrocephalus, intracranial pressure also increases as well as derived from experimental data.

The cranial compartment accounts for about 84% of the total CSF absorption. The spinal absorptive capacity of 16% of the total absorption indicates relatively little role for the spinal compartment in absorption of the accumulated fluid within the CSF space^44^. Thus, in our model, for the sake of simplicity we ignore the role of spinal cord compartment.

## Results

**Figure 6:**
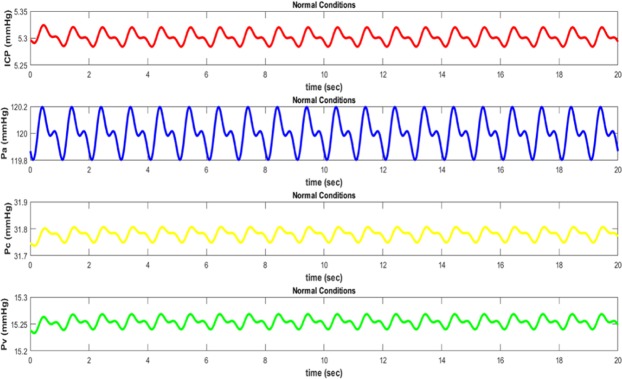
Pressure waveforms in normal conditions (without hydrocephalus): ICP intracranial pressure, Pa arterial pressure, Pc capillary pressure, and Pv venous pressure.

**Figure 7:**
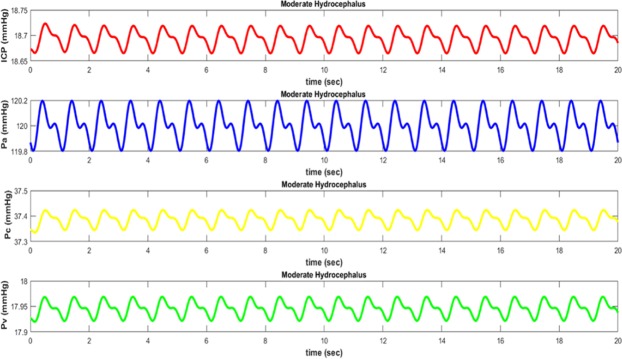
Pressure waveforms in moderate hydrocephalus (*R*_*o*_ = 5 Ω (40 mmHg/ml/min)): ICP intracranial pressure, Pa arterial pressure, *Pc* capillary pressure, and Pv venous pressure.

**Figure 8:**
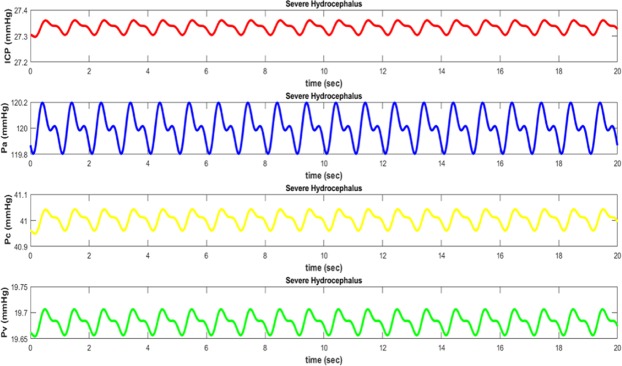
Pressure waveforms in severe hydrocephalus (*R*_*o*_ = 10 Ω (80 mmHg/ml/min)): ICP intracranial pressure, *Pa* arterial pressure, Pc capillary pressure, and Pv venous pressure.

The waveform of arterial, capillary, and venous pressures along with the pressure difference between the arachnoid villi and sagittal sinus in normal conditions have been shown in Fig. 6(a–d) respectively. The waveforms of different degrees of hydrocephalus, i.e. *R*_*o*_ = 5 Ω (40 mmHg/ml/min) and *R*_*o*_ = 10 Ω (80 mmHg/ml/min), are illustrated in Figs 7(a–d) and 8(a–d) respectively. As it is obvious from simulation results, an increasing pressure in different parts of the proposed compartmental model including ventricular system is occurred due to increased resistance of CSF absorption pathway (*R*_*o*_) in case of hydrocephalus. Also, the higher degree of hydrocephalus (larger selected *R*_*o*_ in the model), makes the pressure become greater. However, to simulate the effect of hydrocephalus shunt, we choose the value of *R*_*shunt*_ in proposed model in Fig. 4 so as the amount of ${R}_{o}\parallel {R}_{shunt}$ would be close to the normal resistance that exists against the flow of CSF.

We define shunt efficiency as Eq. (9) in below:

9 $$shunt\,efficiency=(1-\frac{IC{P}_{aftershunting}-IC{P}_{healthybrain}}{IC{P}_{beforeshunting}})\times 100\,$$

**Figure 9:**
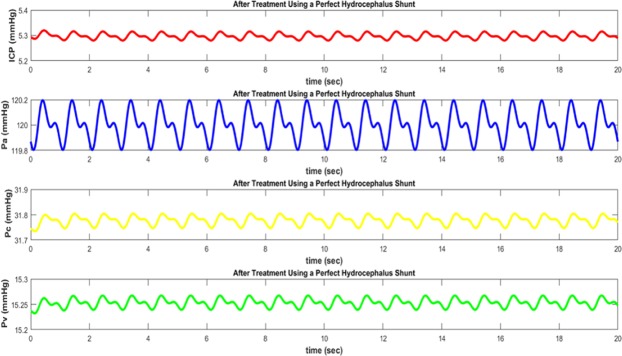
Pressure waveforms in case of using a perfect hydrocephalus shunt: *ICP* intracranial pressure, Pa arterial pressure, Pc capillary pressure, and Pv venous pressure.

By this definition, a shunt with efficiency of 90% has a resistance of about ${R}_{shunt}=1\cdot 25\,{\rm{\Omega }}$ (12 mmHg/ml/min). Using this shunt, the intracranial pressure (ICP) would decrease to 778.892 Pa (5.84 mmHg) (from simulation results) that is close to the normal (healthy brain) ICP. Figure 9(a–d) shows simulation results for a shunt with nearly perfect efficiency, which yields by ${R}_{shunt}=1\cdot 11\,{\rm{\Omega }}a$ (8.88 mmHg/ml/min) In this case, as expected, the simulation results are identical to the normal situation; i.e. using a hydrocephalus shunt, drainage of CSF from the brain ventricular system is well done and patient’s conditions approached to the normal state.

**Figure 10:**
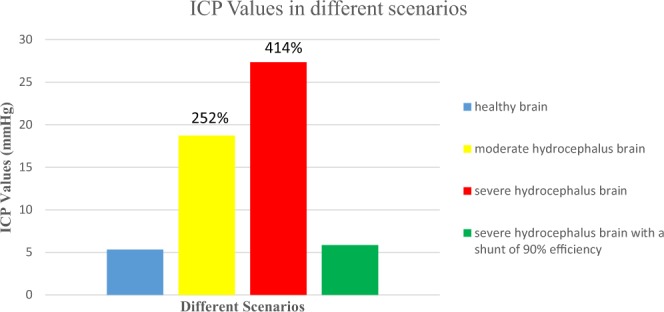
ICP values for four scenarios; from left to right: healthy brain, moderate hydrocephalus brain, severe hydrocephalus brain, and severe hydrocephalus brain with a perfect shunt; increase percent of ICP has been shown in two hydrocephalus scenarios.

ICP values from simulation results have been illustrated in Fig. 10 for four different scenarios; also, ICP’s percent variation compared to the normal scenario is shown in the top of bar curves in Fig. 10 for moderate and severe hydrocephalus. In the moderate hydrocephalus scenario, ICP is 2.496KPa (18.72 mmHg), which shows about 252% increase in the intracranial pressure compare to the normal brain. In the severe hydrocephalus scenario, ICP is 3.648KPa (27.36 mmHg), which shows 414% increase in ICP while by means of a shunt with efficiency of 90% the ICP would be about 778.892 Pa (5.84 mmHg), which only shows an increase of 9.7% compared to the normal scenario. In addition, to compare the shunts with different efficiencies in decreasing ICP, simulation results of ICP for five numbers of assumed values of shunt efficiency have been provided in Table 2; these results have been obtained for the severe hydrocephalus scenario. We simulated various values of *R*_*shunt*_ which are responsible for different shunt efficiencies.

**Table 2:** Simulation results of ICP for five numbers of assumed values of shunt efficiency.

	Shunt1	Shunt2	Shunt3	Shunt4	Shunt5
R_*shunt*_	$1\cdot 11111{\rm{\Omega }}$	$1\cdot 5\,{\rm{\Omega }}$	2 Ω	$2\cdot 5\,{\rm{\Omega }}$	3 Ω
Efficiency	Nearly 100%	95%	89%	84%	80%
ICP	709.13 Pa (5.32 mmHg)	896.131 Pa (6.72 mmHg)	1103.988 Pa (8.28 mmHg)	1282.629 Pa (9.62 mmHg)	1437.8 Pa (10.78 mmHg)

## Discussion

Many theoretical/modeling studies on CSF dynamics were published before the 1970s^45–48^. However, Marmarou was one of the first^21,49^ who integrated CSF production, circulation, absorption, and storage in a suitable theoretical formulation expressed as an electrical model. The mathematical model of cerebrospinal fluid (CSF) pressure volume compensation, introduced by Marmarou in 1973 and modified in later studies, provides a theoretical basis for various diagnosis in hydrocephalus^50^.

In this study, an analytical model, as a modified version of Ursino model, has been provided to simulate hydrocephalus shunt along with the dynamics of CSF production and circulation. In patients with hydrocephalus, the amount of CSF within the brain ventricles is increased and leads to an increase in ICP value in which the brain tissue is severely affected. Various neurological conditions such as intra-ventricular hemorrhage, bleeding in the subarachnoid space, meningitis and congenital absence of the arachnoid villi may cause hydrocephalus. The most common method for hydrocephalus treatment is shunting.

A CSF shunt involves a bypass pathway for drainage of CSF, which accumulated in the brain due to blockage in the path of CSF drainage or the poor functioning of the arachnoid villi. Electrical modeling of hydrocephalus shunting along with CSF dynamics has several advantages. This modeling may lead to a better understanding of the physiology of the disease, especially the changes that occur by the use of a CSF shunt could be observed truly. Electrical model provides a possibility for testing the shunt non-invasively. Also, it provides possibility of checking out a wide range of parameters needed to design a shunt valve before the equipment to be made. Modeling can also help to predict the efficiency of the shunt.

In this paper, an electrical model has been proposed for hydrocephalus shunt that can be used to predict shunt efficiency. In the model, a hydrocephalus shunt can be modeled by a resistance in series with a diode. Shunt parameters such as lumen diameter, material properties, and valve pressure can be combined by a resistance and a diode. This model can potentially be used to test hydrocephalus catheters prior to the *in-vivo* surgical procedure. The method may contribute to the selection of better material and also better physical structure for the shunt that is to be implanted in the patient’s body.

We know that Newtonian fluid flow through a tube assimilates an electronic current flow in a resistor in which the fluid flow is analogous to electrical current and the fluid pressure to voltage. Based on Eq. 9, resistance in the fluid flow results from physical aspects of the tube and the fluid.

Therefore, various shunt configurations with different parameters such as lumen diameter, material properties, port configuration, and valve pressure could be considered in the resistance value. Therefore, using Eq. 9 and knowing the desired values of P and q, we can obtain the optimal R. Then, the surgeon based on the appropriate R derived from the model, as well as the patient’s clinical conditions, obtains the optimal shunt for the patient and thus chooses the best and most suitable shunt for implementation in the patient’s body.

In this study, the value of shunt resistance is assumed linear and time invariant, but shunt resistance may behave nonlinearly and time variant for some reasons such as shunt tube obstruction, disruption of shunt valve control system (irregular opening and closing valve) and reducing the diameter of the shunt tube over time as a result of material deposition. To include these cases in the study, shunt resistance was considered as a non-linear and time variant that represented by Eq. 10.

10 $${R}_{Shunt}=15{{\rm{e}}}^{-0.3{\rm{t}}}(1.2+\,\sin ({\rm{t}}))$$

**Figure 11:**
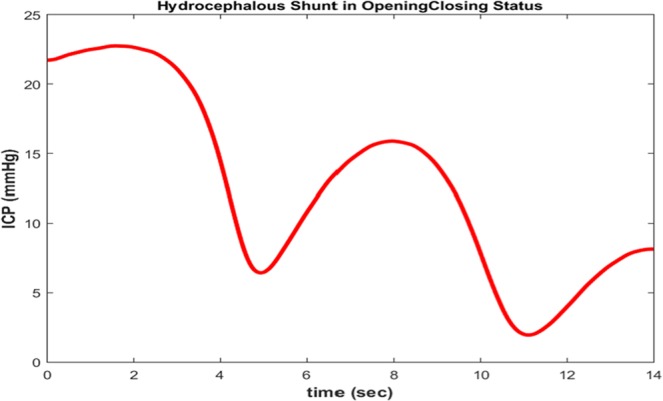
ICP waveform in a manner that hydrocephalous shunt has been considered as a nonlinear and time variant resistance to illustrate over-draining and opening/closing situations of the shunt.

The intracranial pressure (ICP), based on Eq. 10, is shown in Fig. 11.

**Figure 12:**
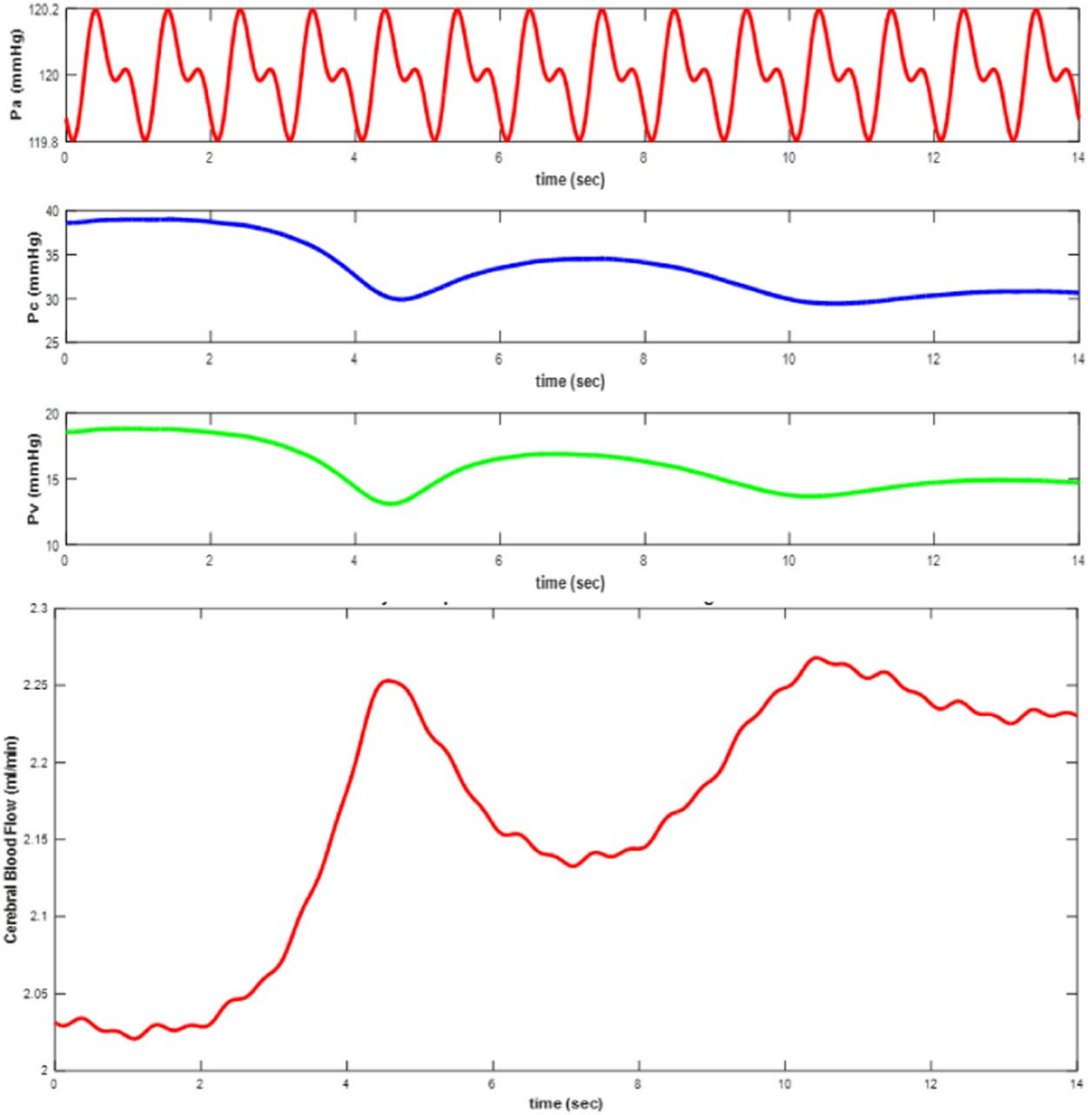
Waveforms of arterial (P_a_), capillary (P_c_), venous (P_v_) pressures and (CBF) cerebral blood flow in a manner that hydrocephalous shunt has been considered as a nonlinear and time variant resistance to illustrate over-draining and opening/closing situations of the shunt.

As shown in Fig. 11, before the time t = 3 s, the pressure of the ICP is greater than 10 mmHg due to the closure of the shunt valve. At interval time, t = 4 s to t = 6 s, the shunt valve is opened and the ICP pressure drops to normal condition. Then, from t = 6 s to t = 9 s, the shunt valve is blocked and the ICP pressure continues to rise. From t > 9 s, the shunt valve opens completely and causes shunt over-draining between t = 10 s to t = 12 s. Figure 12 shows arterial (P_a_), capillary (P_c_), and venous (P_v_) pressures for this conditions.

**Figure 13:**
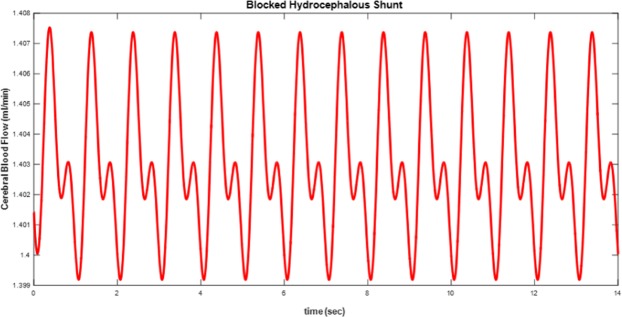
Cerebral blood flow (CBF) in which hydrocephalous shunt is completely blocked (R_shunt_ = 100 Ω).

In Fig. 13, cerebral blood flow (CBF) is shown in which hydrocephalous shunt is completely blocked. In fact, CBF is the current that flow through R_1_ in electrical model in Fig. 5. When the shunt tube is blocked, the CBF decreases.

It is necessary to mention that the model proposed in this paper has some limitations. In our electrical model in this paper, for simplicity we ignored the role of spinal cord compartment, but we suggest that the role of spinal cord compliance in CSF dynamics be considered to achieve a complete model. Also, some literatures reveal that a significant absorption of CSF in subarachnoid space may occur by lymphatic vessels^51,52^. In our model, we did not consider this component that is believed to have a role in CSF absorption. For a complete model, it may be considered. Certainly, experimental studies are required to validate our model.

Although, there are several animal models in which the standard shunts examined and tested, but these models cannot approximate CSF production, postural changes and brain size that happen to human. The postural changes effect on blood pressure consists of a fall as the patient stands up, and a rise when the patient lays down^53^. Thus, postural change effects could be included in the proposed electrical model by inserting a variable component in the voltage supply in Fig. 5. To include the effect of brain size on our model, we can insert a variable capacitor in parallel with *C*_*tissue*_ in the model of Fig. 5 as a larger head has more compliance. All these phenomena may be simulated with an electric model before a shunt being physically implanted in the patient’s brain. In addition, by means of these models the use of animal experiments could be reduced.

## Conclusion

In the treatment of hydrocephalus, a shunt is used to drain additional CSF from the brain ventricles into an elsewhere in the body. In this paper, an electrical model has been proposed for hydrocephalus shunt that can be used to predict shunt efficiency. In our model, we model a hydrocephalus shunt by a resistance in series with a diode. We can combine shunt parameters such as lumen diameter, material properties, and valve pressure by a resistance and a diode. This model can potentially be used to test hydrocephalus catheters prior to the *in-vivo* surgical procedure. The method may contribute to help surgeon to select better material and also better physical structure for the shunt that is to be implanted in the patient’s body.
